# Supplementary figures and images for: Defining the role of the polyasparagine repeat domain of the S. cerevisiae transcription factor Azf1p
Source: PLoS One. 2021 May 21;16(5):e0247285. doi: 10.1371/journal.pone.0247285 (PMC8139511; doi:10.1371/journal.pone.0247285)

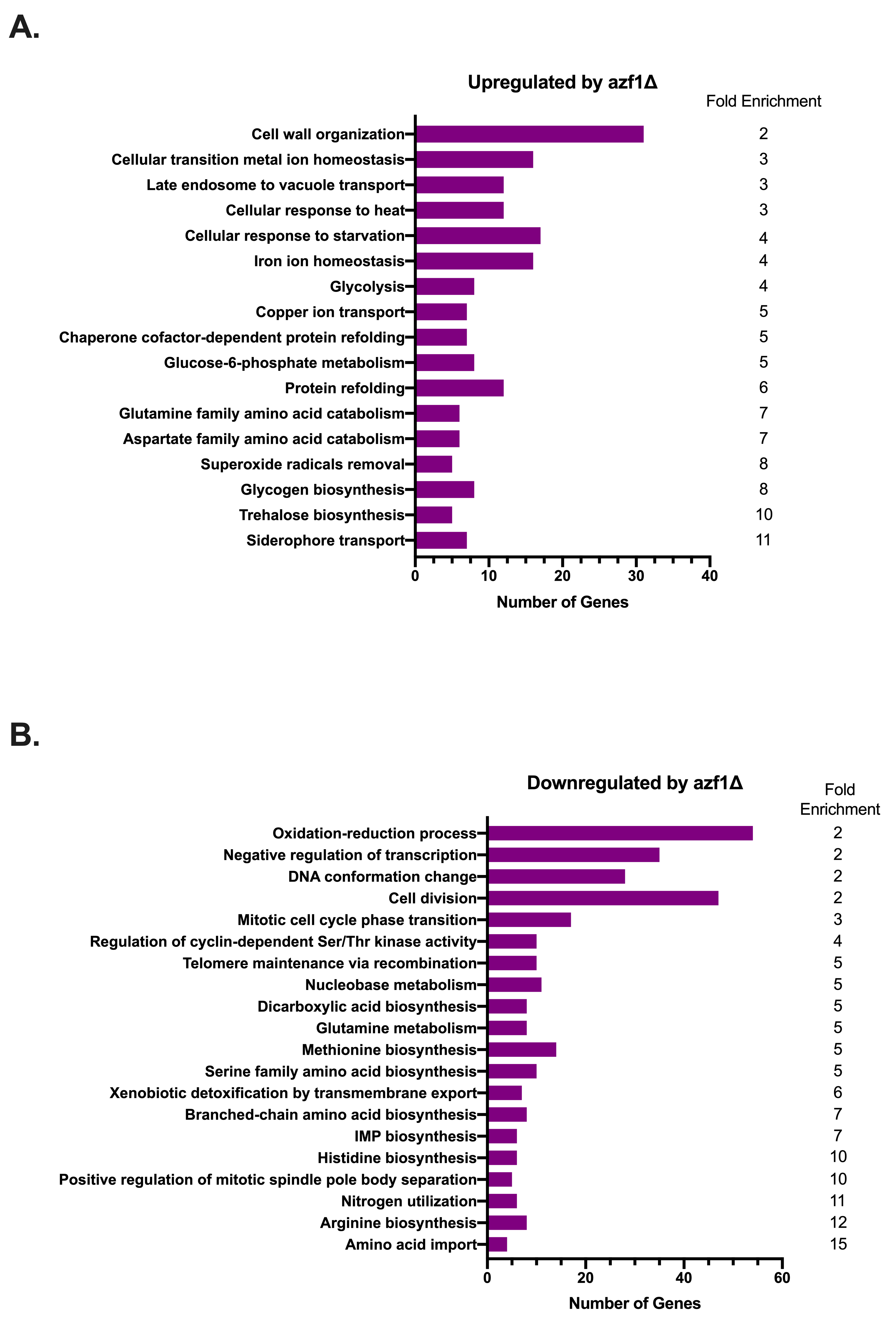

Supplement: S1 Fig — A) GO Enrichment Analysis of genes that are statistically significantly upregulated by azf1Δ (p<0.001) compared to WT as measured by RNA-seq with RNA isolated from cells grown in glucose. B) GO Enrichment Analysis of genes that are statistically significantly downregulated by azf1Δ compared to WT (p<0.001) as measured by RNA-seq using RNA isolated from cells grown in glucose. (TIF) [file pone.0247285.s001.tif]

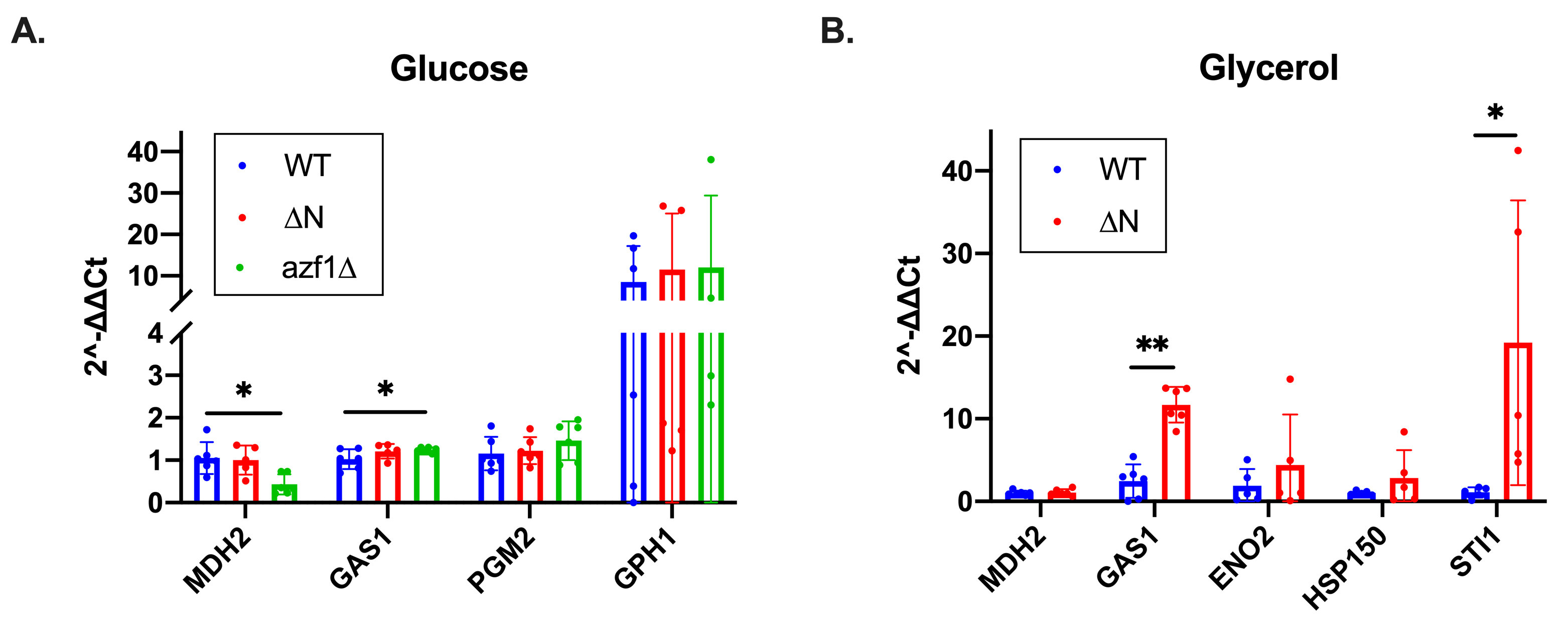

Supplement: S2 Fig — A) Fold change in expression of Azf1p target genes in AZF1ΔN and azf1Δ compared to WT during growth in glucose. B) Fold change in expression of Azf1p target genes in AZF1ΔN compared to WT during growth in glycerol. Error bars represent standard deviation. Statistical significance was calculated using an unpaired student’s t test. * indicates p<0.05 and ** indicates p<0.001. (TIF) [file pone.0247285.s002.tif]

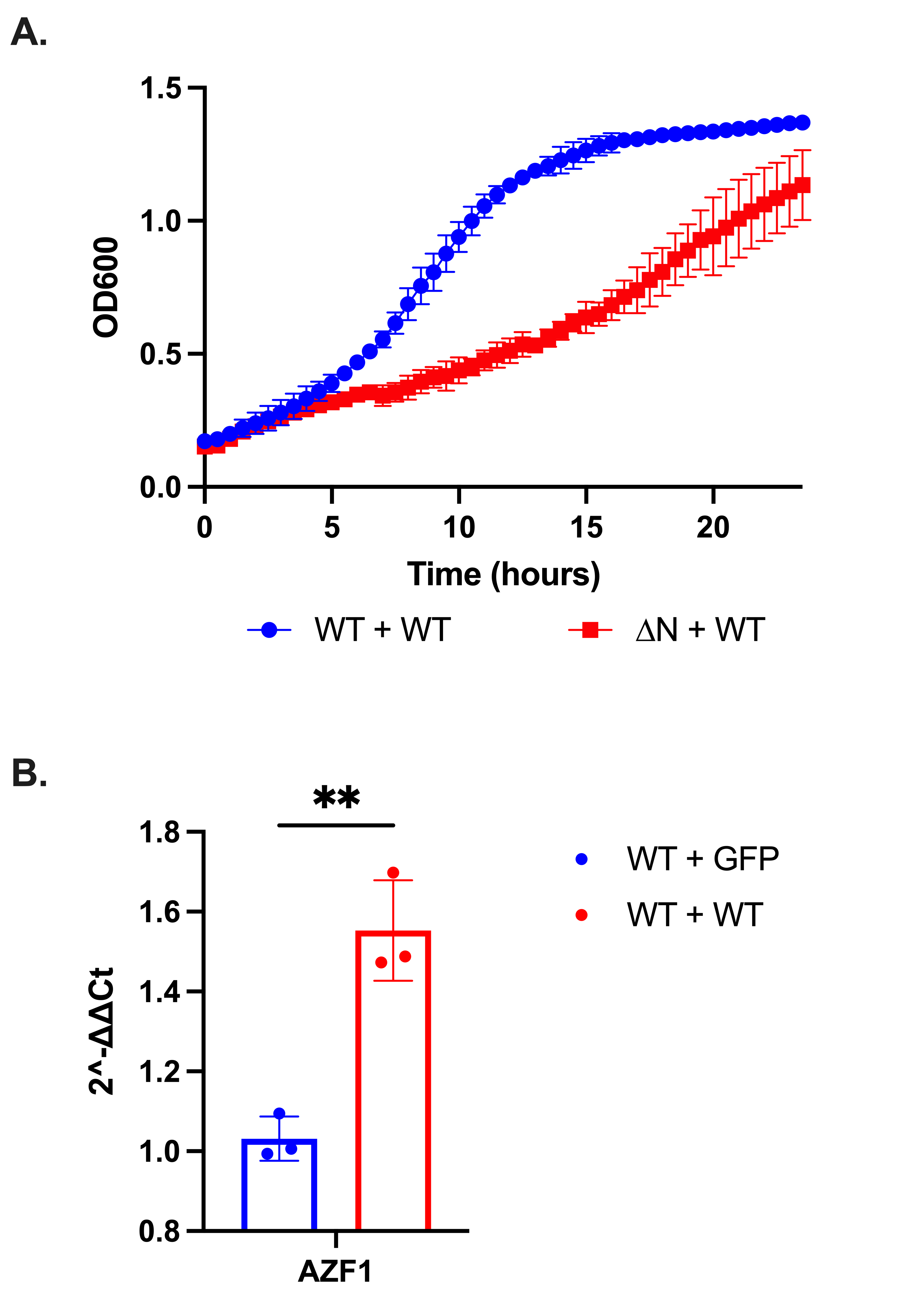

Supplement: S3 Fig — A) Growth of WT (blue) and ΔN (red) cells with glucose as the carbon source. Growth was measured by OD600 in a plate reader for 24 hours. Growth curves are the average of three biological replicates, and error bars indicate standard deviation. B) Fold change in expression of AZF1 in WT cells expressing pBY011-AZF1 compared to pBY011-GFP during growth in glucose. Error bars represent standard deviation. Statistical significance was calculated using an unpaired student’s t test (p = 0.0028). (TIF) [file pone.0247285.s003.tif]
