# Supplementary material for: Defining the role of the polyasparagine repeat domain of the S. cerevisiae transcription factor Azf1p
Source: PLoS One. 2021 May 21;16(5):e0247285. doi: 10.1371/journal.pone.0247285 (PMC8139511; doi:10.1371/journal.pone.0247285)
Supplement: S1 Table — (PDF) [file pone.0247285.s004.pdf]

S1 Table. Plasmids used in this work.

Plasmid	Description	Source
pBY011-AZF1	*AZF1* is under the control of the GAL1/10 promoter	Harvard PlasmID
pBY011-AZF1ΔN	*AZF1ΔN* is under the control of the GAL1/10 promoter	This work
pML104-polyQ	Cas9 and a guide RNA sequence targeting the polyQ domain	Laughery et al. 2015; This work
