# Supplementary material for: Defining the role of the polyasparagine repeat domain of the S. cerevisiae transcription factor Azf1p
Source: PLoS One. 2021 May 21;16(5):e0247285. doi: 10.1371/journal.pone.0247285 (PMC8139511; doi:10.1371/journal.pone.0247285)
Supplement: S2 Table — (PDF) [file pone.0247285.s005.pdf]

**S2 Table. Primers used in this work.**

| Sequence | Description |
|---|---|
| CCACGGACGCCTCTATCAAC | Forward primer for amplification of the polyN and polyQ domains of *AZF1* |
| CACTGCCTTCAATTACGTCG | Reverse primer for amplification of the polyN and polyQ domains of *AZF1* |
| ATATACTTCAAAAGATTCAGATATTGAAAAATTACCAGCCAGTGCACCATACCACAGC | Forward primer for amplification of *URA3* (promoter, ORF, & terminator) from pRS416-tetO7-GFP with 5' homology to *AZF1* |
| AAACTCGAAAAAAAAAAAAAGGTAGTGCCTCTAAGGCCTGACCGCATAGGGTAATAACTG | Reverse primer for amplification of *URA3* (promoter, ORF, & terminator) from pRS416-tetO7-GFP with 3' homology to *AZF1* |
| ATTCAGACTCCTGGGATACG | Forward primer for amplification of *azf1-Δ1::URA3* junction; anneals 5' of *AZF1* locus |
| TTCTGTGCAGTTGGGTTAAG | Reverse primer for amplification of *azf1-Δ1::URA3* junction; anneals internal to *URA3* promoter |
| ATGATGGCAATGACAGTAGCAGTATTAATTCCGCCACCAG | Forward primer for QuikChange deletion of the polyN domain of *AZF1* |
| CTGGTGGCGGAATTAATACTGCTACTGTCATTGCCATCATC | Reverse primer for QuikChange deletion of the polyN domain of *AZF1* |
| AGGCCAAGAACGGTAAAGGT | Forward primer for RT-qPCR of *MDH2* |
| TAATTGATCTGCCCCAGGAG | Reverse primer for RT-qPCR of *MDH2* |
| ACTTCCGCCAACACAAAGTC | Forward primer for RT-qPCR of *GAS1* |
| ACGTCATCGGAAACAACACA | Reverse primer for RT-qPCR of *GAS1* |
| ATGTCCCTATTGTCATCGGC | Forward primer for RT-qPCR of *AZF1* |
| TCACCTGTGTGTAGTCGTTC | Reverse primer for RT-qPCR of *AZF1* |

| | |
|---|---|
| CCTTCAACGTTCCAGCCTTC | Forward primer for RT-qPCR of *ACT1* |
| CCGGCGTAAATTGGAACAAC | Reverse primer for RT-qPCR of *ACT1* |
| CAGGCACAGCCTGATTTAGG | Forward primer for RT-qPCR of *STI1* |
| ATCTTGGCCAATAGCTTGCG | Reverse primer for RT-qPCR of *STI1* |
| ACTGGACCATACGGTAAGGC | Forward primer for RT-qPCR of *PGM2* |
| ACCATCGGATGCAGCACC | Reverse primer for RT-qPCR of *PGM2* |
| CAGCCAGCCAAGTAAGCG | Forward primer for RT-qPCR of *HSP150* |
| TTGTGGTGGTGGACCGTC | Reverse primer for RT-qPCR of *HSP150* |
| GGAAAGAGTTCTTGCTGTCGCG | Forward primer for RT-qPCR of *GPH1* |
| TAGACTCTGCGCGTTGTTGC | Reverse primer for RT-qPCR of *GPH1* |
| CGGTGGTTCCCACGCTG | Forward primer for RT-qPCR of *ENO1* |
| CCTTCGTCACCGACGTTACC | Reverse primer for RT-qPCR of *ENO1* |
