# Supplementary material for: Defining the role of the polyasparagine repeat domain of the S. cerevisiae transcription factor Azf1p
Source: PLoS One. 2021 May 21;16(5):e0247285. doi: 10.1371/journal.pone.0247285 (PMC8139511; doi:10.1371/journal.pone.0247285)
Supplement: S3 Table — (PDF) [file pone.0247285.s006.pdf]

S3 Table. Yeast strains used in this work.

Strain	Genotype	Description	Source
WT	*MATa his3Δ1 leu2Δ0 met15Δ0 ura3Δ0 AZF1-GFP*	BY4741 background in which AZF1 is C-terminally tagged with GFP	Hu *et al.* 2003, Howson *et al.* 2005
azf1Δ	BY4741 azf1-Δ1::URA3	URA3 is inserted between the AZF1 ORF and its promoter, abolishing expression of AZF1	This work
AZF1ΔN	BY4741 AZF1ΔN-GFP	BY4741 AZF1-GFP in which the polyN domain of AZF1 is deleted	This work
