# Supplementary material for: Defining the role of the polyasparagine repeat domain of the S. cerevisiae transcription factor Azf1p
Source: PLoS One. 2021 May 21;16(5):e0247285. doi: 10.1371/journal.pone.0247285 (PMC8139511; doi:10.1371/journal.pone.0247285)
Supplement: S4 Table — (PDF) [file pone.0247285.s007.pdf]

S4 Table. Fold change in *AZF1* expression in 3 biological replicates of *azf1Δ* compared to WT.

Sample	2^-ΔΔCt
WT	1
azf1Δ 1	1.51E-03
azf1Δ 2	1.53E-03
azf1Δ 3	8.69E-03
