# Supplementary material for: Defining the role of the polyasparagine repeat domain of the S. cerevisiae transcription factor Azf1p
Source: PLoS One. 2021 May 21;16(5):e0247285. doi: 10.1371/journal.pone.0247285 (PMC8139511; doi:10.1371/journal.pone.0247285)
Supplement: Attachment — Submitted filename: ResponsetoReviewers2.pdf [file pone.0247285.s012.pdf]

Response to Reviewers:

Reviewer #1: (No Response)

Reviewer #2: The authors addressed all comments, but I still have two minor concerns:

-I could not see the new figure S3 because, as the other supplementary figures, was not loaded

We have uploaded new versions of the supplemental figures (which include the additional data asked for below). The manuscript should now be complete.

-The authors expressed AZF1 from a plasmid where the gene is under control of GAL promoter. However, by qPCR, they observed expression of the gene also in glucose and this determined the rescue of the phenotype. It would be interesting to know the expression level of azf1 in glucose so I would add in the figure s3 the results of qPCR because, although it is known that gal promoter is leaky, the use of it to express protein in medium with glucose is not common.

We have added the qPCR data as reviewer 2 suggested to Figure S3B. We agree, this is not common, but the best we were able to use under our current working circumstances.
