# Supplementary material for: Defining the role of the polyasparagine repeat domain of the S. cerevisiae transcription factor Azf1p
Source: PLoS One. 2021 May 21;16(5):e0247285. doi: 10.1371/journal.pone.0247285 (PMC8139511; doi:10.1371/journal.pone.0247285)
Supplement: Attachment — Submitted filename: ResponsetoReviewers.docx [file pone.0247285.s011.docx]

Response to Reviewers:

Reviewer #1:

*This manuscript presents an analysis of the role of the poly-asparagine (polyN) domain of the yeast protein Azf1p. The authors report a role of the polyN domain in mediating Azf1p's function as a transcriptional regulator of carbon metabolism. They show that the polyN domain is not required for prion-like formation in yeast and provide a bioinformatics analysis across diverse yeast strains revealing diversity in length of the repeat across different yeast strains.*

*Overall the experiments are performed to a high standard and are rigorous and the conclusions are supported by the data. I therefore recommend publication of this study in PLoSOne because it fulfills the requirements for publication in this journal.*

Many thanks

*I have one suggestion for the authors to consider. They may want to to perform rescue experiments with WT Azf1p (e.g., from a plasmid) to make sure the growth phenotypes and the changes in gene expression that they report in their ∆N mutants are definitely caused by the mutation and not some other perturbation.*

This was a fantastic suggestion by Reviewer 1 and one which we spent the past several weeks trying to implement. We have included now an additional figure (S3 Fig) and updated the text to show that indeed the growth phenotypes are caused by the ∆N mutations.

Reviewer #2:

*In the present paper by Stewart et al, the authors analyze the function of azf1 protein, a Zinc-finger transcription factor, regulating the expression of genes involved in growth, carbon metabolism and in cell wall integrity depending on carbon source present in growth medium. The protein contains polyN and polyQ domain typical of prions. Indeed it has been shown that azf1 exists in prion state and confer resistance to the drug radicicol. The aim of the paper is to characterize the polyN domain of azf1 and define its role in transcription and prion formation. The authors demonstrate that this domain has a subtle role in transcription but not in prion formation. Investigations on yeast prion has an important spill-over on the improvement of yeast strains for fermentation applications and small molecule production.*

*The paper is well written and clear and experiments are carefully designed and mapped out. Moreover Data are convincing, well presented and discussed.*

Much appreciated

*I have only a minor drawbacks that need to be addressed before publication.
1- Pag 4 at the end of introduction “cell well integrity” must be corrected in “cell wall integrity”*

Fixed

*2- Pag 10 paragraph “Differential roles of the polyN domain during growth in different carbon sources”:
The concept expressed in line 3-4 referring to the phenotype of azf1delta strain is then repeated in line 6 with the sentence “ Consistent with previous reports, azf1∆ does not support growth on glycerol”. I think that the latter sentence can be omitted.*

Also corrected in the text
